# Bis(nona­fluoro­butane­sulfonato-κ*O*)dioxidotris(tetra­hydro­furan-κ*O*)uranium(VI)

**DOI:** 10.1107/S1600536807065865

**Published:** 2007-12-12

**Authors:** Koichiro Takao, Yasuhisa Ikeda

**Affiliations:** a Research Laboratory for Nuclear Reactors, Tokyo Institute of Technology, 2-12-1-N1-34, O-okayama, Meguro-ku, Tokyo 152-8550, Japan

## Abstract

In the title compound, [U(C_4_F_9_O_3_S)O_2_(C_4_H_8_O)_3_], each U^VI^ ion is located on a twofold rotation axis and is seven-coordinated by two terminal O atoms in the axial positions [U—O = 1.737 (5) Å] and five O atoms from two monodentate nona­fluoro­butane­sulfonate (NfO^−^) and three tetra­hydro­furan ligands in the equatorial plane [U—O = 2.388 (5)–2.411 (4) Å] in a penta­gonal–bipyramidal geometry. The crystal packing exhibits weak inter­molecular C—H⋯O hydrogen bonds involving the non-coordinated O atoms of the NfO^−^ ligands.

## Related literature

For related crystal structures, see: Alcock *et al.* (1993 ▶); Berthet *et al.* (2000 ▶); Charpin *et al.* (1987 ▶); Oldham *et al.* (2006 ▶); Rebizant *et al.* (1987 ▶); Thuéry *et al.* (1995 ▶); Wilkerson *et al.* (1999 ▶).
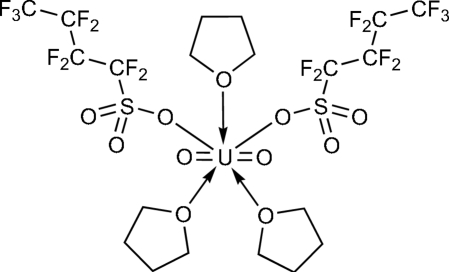


## Experimental

### 

#### Crystal data

[U(C_4_F_9_O_3_S)O_2_(C_4_H_8_O)_3_]
*M* *_r_* = 1084.54
Monoclinic, 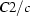

*a* = 23.803 (12) Å
*b* = 11.197 (5) Å
*c* = 12.919 (5) Å
β = 101.46 (4)°
*V* = 3375 (3) Å^3^
*Z* = 4
Mo *K*α radiation
μ = 5.08 mm^−1^
*T* = 93 (2) K
0.50 × 0.30 × 0.20 mm

#### Data collection

Rigaku R-AXIS RAPID diffractometer
Absorption correction: numerical (**NUMABS**; Higashi, 1999 ▶) *T*_min_ = 0.185, *T*_max_ = 0.430
12094 measured reflections
3839 independent reflections
3492 reflections with *I* > 2σ(*I*)
*R*_int_ = 0.079

#### Refinement

*R*[*F* ^2^ > 2σ(*F* ^2^)] = 0.046
*wR*(*F* ^2^) = 0.125
*S* = 1.13
3839 reflections
237 parameters
H-atom parameters constrained
Δρ_max_ = 1.64 e Å^−3^
Δρ_min_ = −2.31 e Å^−3^

### 

Data collection: *PROCESS-AUTO* (Rigaku, 2006 ▶); cell refinement: *PROCESS-AUTO*; data reduction: *CrystalStructure* (MSC/Rigaku, 2006 ▶); program(s) used to solve structure: *SIR92* (Altomare *et al.*, 1994 ▶); program(s) used to refine structure: *SHELXL97* (Sheldrick, 1997 ▶); molecular graphics: *ORTEP-3* (Farrugia, 1997 ▶); software used to prepare material for publication: *CrystalStructure*.

## Supplementary Material

Crystal structure: contains datablocks I, global. DOI: 10.1107/S1600536807065865/cv2371sup1.cif

Structure factors: contains datablocks I. DOI: 10.1107/S1600536807065865/cv2371Isup2.hkl

Additional supplementary materials:  crystallographic information; 3D view; checkCIF report

## Figures and Tables

**Table 1:** Hydrogen-bond geometry (Å, °)

*D*—H⋯*A*	*D*—H	H⋯*A*	*D*⋯*A*	*D*—H⋯*A*
C6—H6*B*⋯O4^i^	0.99	2.63	3.57 (1)	159
C7—H7*A*⋯O3^ii^	0.99	2.47	3.409 (9)	158

Symmetry codes: (i) 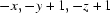; (ii) 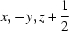.

**Fig. 1.**
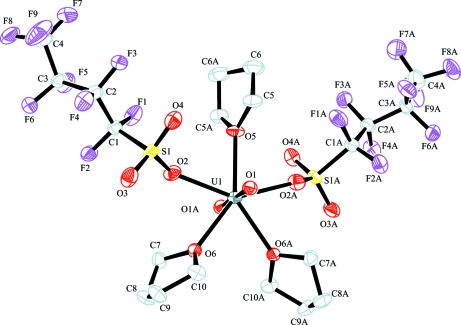
Molecular structure of I showing the atomic numbering and 50% probability displacement ellipsoids [symmetry code: (A) -x, +y, -z + 3/2. Hydrogen atoms omitted for clarity.

**Fig. 2.**
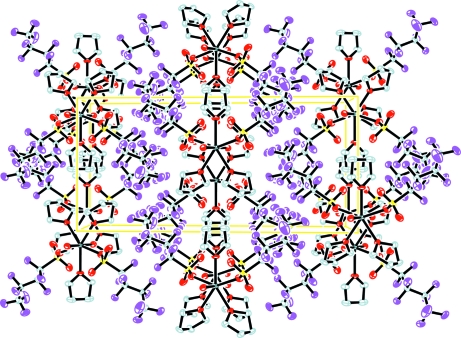
Packing diagram of I viewed along c axis. Hydrogen atoms are omitted for clarity.

**Fig. 3.**
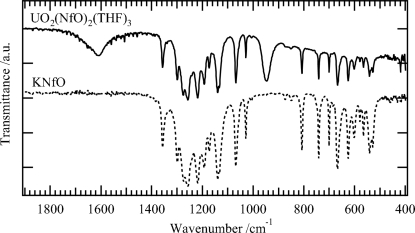
IR spectra of I (solid line) and KNfO (dashed line) in KBr.

## supplementary crystallographic information

### Comment

In preparation of uranyl(VI) compounds, a source of uranyl(VI) ion should contain only easily exchangeable ligands. In most cases, uranyl(VI) nitrate or chloride hydrates are convenient for this purpose. However, in syntheses of water-sensitive uranyl(VI) compounds, for instance alkoxides and amides, use of anhydrous starting materials must be required. To solve this problem, Berthet and co-workers proposed a preparation method of an anhydrous uranyl(VI) trifluoromethanesulfonate (UO_2_(TfO)_2_) from a reaction of UO_3_ with trifluoromethanesulfonic anhydride (Berthet *et al.* 2000).

We followed and modified Berthet's process for preparation of a novel anhydrous uranyl(VI) salt. We tried to synthesize uranyl(VI) nonafluorobutanesulfonate from UO_3_ and nonafluorobutanesulfonic anhydride. The obtained crystals from tetrahydrofuran (THF) solution were of the title compound, UO_2_(NfO)_2_(THF)_3_ (I, NfO^-^ = *n*-C_4_F_9_SO_3_^-^). In this paper, we report its structure.

The molecular structure and packing diagram of I are shown in Figs. 1 and 2, respectively. The U atom in I is surrounded by seven O atoms; two O are at the axial positions, and the remaining five O from NfO^-^ and THF are in the equatorial plane. As a result, the coordination geometry around U in I is pentagonal bipyramidal. Two NfO^-^ anions in I are unidentate and non-adjacent. The O_NfO_–U–O_THF_ and O_THF_–U–O_THF_ bond angles are almost equal to 72°. Furthermore, the O_NfO_···O_THF_ and O_THF_···O_THF_ distances are quite similar to each other [mean: 2.82 (2) Å]. In addition, deviations of the coordinating O atoms in NfO^-^ and THF from the mean equatorial plane are small (< 0.07 Å). These results indicate that the equatorial plane is close to the ideal pentagon. Bond length between U and the axial O is 1.737 (5) Å, which is comparable with most uranyl(VI) compounds. The U–O_NfO_ bond length (2.388 (5) Å) is identical to those found in other uranyl(VI) complexes with alkylated and perfluoroalkylated sulfonates (Oldham *et al.* 2006, Berthet *et al.* 2000, Thuéry *et al.* 1995, Alcock *et al.* 1993). The average U–O_THF_ bond length is 2.41 (1) Å, which is similar to those in the reported uranyl–THF complexes (Oldham *et al.* 2006, Rebizant *et al.* 1987, Wilkerson *et al.* 1999, Charpin *et al.* 1987). Intermolecular short contacts are observed between C–H of THF and the non-coordinating O of NfO^-^. The C···O distances are 3.57 and 3.41 Å, and the C–H···O angles are 158.7 and 158.0° as summarized in Table 2. Therefore, these contacts can be regarded as weak intermolecular hydrogen bonds.

Figure 3 shows the IR spectrum of I (solid line) in KBr together with that of a potassium salt of NfO^-^ (KNfO, dashed line). Characteristic asymmetric stretching of UO_2_^2+^ was observed at 947 cm^-1^. Other spectral feature except for the band at 1610 cm^-1^ shows good agreement with that of KNfO, indicating the presence of NfO^-^ in I. The peak at 1610 cm^-1^ may be due to the coordinated THF.

As described above, the anhydrous uranyl(VI) source must be needed in the preparation of the water-sensitive uranyl(VI) compounds. We believe that compound I provides an alternative selection of the anhydrous uranyl(VI) salts as the starting material.

The related crystal structures were published - UO_2_(TfO)_2_(THF)_3_ (Oldham *et al.* 2006), UO_2_(TfO)_2_(pyridine)_3_ (Berthet *et al.* 2000), UO_2_(TfO)_2_(OH_2_)_3._benzo-15-crown -5 (Thuéry *et al.* 1995), UO_2_(RSO_3_)_2_(OH_2_) (*R* = Et, *p*-tolyl, mesityl; Alcock *et al.* 1993), UO_2_Br_2_(THF)_3_ (Rebizant *et al.* 1987), UO_2_Cl_2_(THF)_3_ (Wilkerson *et al.* 1999), and [UO_2_Cl(THF)_2_]_2_(µ-Cl)_2_ (Charpin *et al.* 1987).

### Experimental

All manipulations for the preparation of the title compound I were performed in a glove box filled by dry argon gas. Uranium(VI) trioxide (0.37 g) was mixed with nonafluorobutanesulfonic anhydride (Nf_2_O, 5.0 g) in a round-bottom flask. The mixture was refluxed under the inert atmosphere for 24 h, and then volatiles were evaporated under reduced pressure. The greenish-yellow residue was dissolved in tetrahydrofuran (THF, 2 ml), and gently warmed. After storing the THF solution at room temperature overnight, the yellow crystals of I deposited. This compound is highly hygroscopic. It should be kept in the THF mother liquor or paraffin oil, and immediately immersed in the cold nitrogen gas flow after mounting on the glass fiber.

IR spectra of I and KNfO in KBr were measured by SHIMADZU FTIR-8400S equipped with a diffuse reflectance attachment.

### Refinement

All hydrogen atoms were geometrically positioned (C—H 0.99 Å) and refined as riding on their parent atoms, with *U*_iso_(H) = 1.2*U*_eq_(C).

### Crystal data

**Table:**

[U(C_4_F_9_O_3_S)O_2_(C_4_H_8_O)_3_]	*F*_000_ = 2072
*M**_r_* = 1084.54	*D*_x_ = 2.135 Mg m^−^^3^
Monoclinic, *C*2/*c*	Mo *K*α radiation λ = 0.71075 Å
Hall symbol: -C 2yc	Cell parameters from 17062 reflections
*a* = 23.803 (12) Å	θ = 3.2–27.5º
*b* = 11.197 (5) Å	µ = 5.08 mm^−^^1^
*c* = 12.919 (5) Å	*T* = 93 (2) K
β = 101.46 (4)º	Block, yellow
*V* = 3375 (3) Å^3^	0.50 × 0.30 × 0.20 mm
*Z* = 4	

### Data collection

**Table:**

Rigaku R-AXIS RAPID diffractometer	3839 independent reflections
Radiation source: fine-focus sealed tube	3492 reflections with *I* > 2σ(*I*)
Monochromator: graphite	*R*_int_ = 0.079
Detector resolution: 10.00 pixels mm^-1^	θ_max_ = 27.5º
*T* = 93(2) K	θ_min_ = 3.2º
ω scans	*h* = −30→30
Absorption correction: numerical(*NUMABS*; Higashi, 1999)	*k* = −13→14
*T*_min_ = 0.185, *T*_max_ = 0.430	*l* = −16→16
12094 measured reflections	

### Refinement

**Table:**

Refinement on *F*^2^	Secondary atom site location: difference Fourier map
Least-squares matrix: full	Hydrogen site location: inferred from neighbouring sites
*R*[*F*^2^ > 2σ(*F*^2^)] = 0.046	H-atom parameters constrained
*wR*(*F*^2^) = 0.125	*w* = 1/[σ^2^(*F*_o_^2^) + (0.0574*P*)^2^ + 32.741*P*] where *P* = (*F*_o_^2^ + 2*F*_c_^2^)/3
*S* = 1.13	(Δ/σ)_max_ < 0.001
3839 reflections	Δρ_max_ = 1.64 e Å^−^^3^
237 parameters	Δρ_min_ = −2.31 e Å^−^^3^
Primary atom site location: structure-invariant direct methods	Extinction correction: none

### Special details

**Table:**

Geometry. All e.s.d.'s (except the e.s.d. in the dihedral angle between two l.s. planes) are estimated using the full covariance matrix. The cell e.s.d.'s are taken into account individually in the estimation of e.s.d.'s in distances, angles and torsion angles; correlations between e.s.d.'s in cell parameters are only used when they are defined by crystal symmetry. An approximate (isotropic) treatment of cell e.s.d.'s is used for estimating e.s.d.'s involving l.s. planes.
Refinement. Refinement of *F*^2^ against ALL reflections. The weighted *R*-factor *wR* and goodness of fit *S* are based on *F*^2^, conventional *R*-factors *R* are based on *F*, with *F* set to zero for negative *F*^2^. The threshold expression of *F*^2^ > 2σ(*F*^2^) is used only for calculating *R*-factors(gt) *etc*. and is not relevant to the choice of reflections for refinement. *R*-factors based on *F*^2^ are statistically about twice as large as those based on *F*, and *R*– factors based on ALL data will be even larger.

### Fractional atomic coordinates and isotropic or equivalent isotropic displacement parameters (Å^2^)

**Table:**

	*x*	*y*	*z*	*U*_iso_*/*U*_eq_	
U1	0.0000	0.12170 (3)	0.7500	0.02259 (12)	
S1	0.09505 (8)	0.22653 (15)	0.58244 (13)	0.0294 (3)	
F1	0.1486 (2)	0.3833 (4)	0.7156 (4)	0.0422 (11)	
F2	0.20365 (19)	0.2469 (4)	0.6672 (4)	0.0395 (10)	
F3	0.13671 (19)	0.4996 (4)	0.5399 (4)	0.0419 (10)	
F4	0.1690 (2)	0.3527 (4)	0.4567 (3)	0.0398 (10)	
F5	0.2474 (2)	0.4856 (4)	0.6850 (4)	0.0464 (11)	
F6	0.2730 (2)	0.3752 (3)	0.5638 (4)	0.0390 (10)	
F7	0.2196 (3)	0.6645 (5)	0.5544 (7)	0.080 (2)	
F8	0.3022 (2)	0.5971 (5)	0.5425 (5)	0.0563 (14)	
F9	0.2304 (3)	0.5585 (7)	0.4199 (5)	0.081 (2)	
O1	−0.0415 (2)	0.1211 (4)	0.6231 (4)	0.0282 (10)	
O2	0.0820 (2)	0.1855 (4)	0.6840 (4)	0.0335 (11)	
O3	0.1141 (3)	0.1326 (4)	0.5233 (5)	0.0417 (13)	
O4	0.0536 (2)	0.3089 (5)	0.5265 (4)	0.0358 (11)	
O5	0.0000	0.3367 (5)	0.7500	0.0299 (14)	
O6	0.0527 (2)	−0.0532 (4)	0.7166 (3)	0.0241 (9)	
C1	0.1587 (3)	0.3193 (6)	0.6320 (5)	0.0279 (13)	
C2	0.1734 (3)	0.4081 (6)	0.5496 (6)	0.0318 (14)	
C3	0.2351 (3)	0.4587 (6)	0.5806 (6)	0.0312 (14)	
C4	0.2464 (4)	0.5727 (8)	0.5205 (8)	0.051 (2)	
C5	−0.0373 (4)	0.4110 (6)	0.6716 (5)	0.0350 (16)	
H5A	−0.0775	0.3835	0.6607	0.042*	
H5B	−0.0246	0.4094	0.6031	0.042*	
C6	−0.0309 (4)	0.5352 (7)	0.7194 (7)	0.050 (2)	
H6A	−0.0582	0.5480	0.7670	0.060*	
H6B	−0.0370	0.5974	0.6639	0.060*	
C7	0.1132 (3)	−0.0750 (6)	0.7639 (5)	0.0309 (14)	
H7A	0.1196	−0.0711	0.8419	0.037*	
H7B	0.1382	−0.0158	0.7386	0.037*	
C8	0.1247 (4)	−0.1995 (6)	0.7276 (6)	0.0368 (16)	
H8A	0.1657	−0.2103	0.7255	0.044*	
H8B	0.1130	−0.2609	0.7743	0.044*	
C9	0.0880 (3)	−0.2052 (6)	0.6184 (6)	0.0361 (16)	
H9A	0.0789	−0.2888	0.5966	0.043*	
H9B	0.1073	−0.1663	0.5661	0.043*	
C10	0.0343 (3)	−0.1371 (6)	0.6301 (5)	0.0303 (14)	
H10A	0.0179	−0.0940	0.5641	0.036*	
H10B	0.0049	−0.1925	0.6470	0.036*	

### Atomic displacement parameters (Å^2^)

**Table:**

	*U*^11^	*U*^22^	*U*^33^	*U*^12^	*U*^13^	*U*^23^
U1	0.0297 (2)	0.01924 (17)	0.01910 (17)	0.000	0.00560 (12)	0.000
S1	0.0323 (9)	0.0291 (8)	0.0281 (7)	−0.0095 (7)	0.0091 (6)	−0.0037 (6)
F1	0.048 (3)	0.048 (3)	0.033 (2)	−0.019 (2)	0.015 (2)	−0.0128 (18)
F2	0.029 (2)	0.038 (2)	0.048 (2)	−0.0002 (18)	−0.0007 (18)	0.0190 (19)
F3	0.033 (2)	0.030 (2)	0.063 (3)	0.0040 (17)	0.010 (2)	0.011 (2)
F4	0.043 (3)	0.049 (2)	0.027 (2)	−0.008 (2)	0.0061 (18)	0.0002 (18)
F5	0.044 (3)	0.048 (3)	0.046 (2)	−0.018 (2)	0.006 (2)	−0.009 (2)
F6	0.031 (2)	0.033 (2)	0.054 (3)	0.0030 (17)	0.011 (2)	0.0047 (18)
F7	0.058 (4)	0.032 (2)	0.156 (7)	0.003 (3)	0.037 (4)	0.025 (3)
F8	0.040 (3)	0.046 (3)	0.082 (4)	−0.014 (2)	0.010 (3)	0.022 (3)
F9	0.069 (4)	0.107 (5)	0.061 (4)	−0.032 (4)	−0.002 (3)	0.050 (4)
O1	0.031 (3)	0.024 (2)	0.033 (2)	0.0025 (18)	0.015 (2)	0.0033 (17)
O2	0.044 (3)	0.031 (2)	0.028 (2)	−0.002 (2)	0.014 (2)	0.0048 (19)
O3	0.042 (3)	0.039 (3)	0.049 (3)	−0.014 (2)	0.020 (3)	−0.017 (2)
O4	0.033 (3)	0.043 (3)	0.030 (2)	−0.006 (2)	0.003 (2)	0.006 (2)
O5	0.038 (4)	0.016 (3)	0.032 (3)	0.000	−0.003 (3)	0.000
O6	0.025 (2)	0.023 (2)	0.026 (2)	−0.0016 (17)	0.0078 (17)	−0.0046 (17)
C1	0.031 (4)	0.027 (3)	0.026 (3)	−0.002 (3)	0.008 (3)	−0.001 (2)
C2	0.030 (4)	0.027 (3)	0.037 (4)	−0.003 (3)	0.004 (3)	0.002 (3)
C3	0.025 (3)	0.027 (3)	0.040 (4)	−0.006 (3)	0.002 (3)	0.003 (3)
C4	0.040 (5)	0.035 (4)	0.078 (6)	−0.006 (3)	0.010 (4)	0.022 (4)
C5	0.049 (5)	0.027 (3)	0.029 (3)	0.002 (3)	0.008 (3)	0.006 (3)
C6	0.070 (6)	0.026 (3)	0.058 (5)	0.005 (4)	0.024 (5)	0.002 (3)
C7	0.025 (3)	0.034 (3)	0.033 (3)	0.000 (3)	0.004 (3)	−0.006 (3)
C8	0.041 (4)	0.026 (3)	0.041 (4)	0.013 (3)	0.003 (3)	0.004 (3)
C9	0.042 (4)	0.033 (3)	0.034 (4)	0.011 (3)	0.008 (3)	−0.005 (3)
C10	0.034 (4)	0.030 (3)	0.023 (3)	0.002 (3)	−0.003 (3)	−0.005 (2)

### Geometric parameters (Å, °)

**Table:**

U1—O1	1.737 (5)	O6—C10	1.460 (7)
U1—O1^i^	1.737 (5)	O6—C7	1.468 (8)
U1—O2	2.388 (5)	C1—C2	1.546 (9)
U1—O2^i^	2.388 (5)	C2—C3	1.550 (10)
U1—O5	2.407 (6)	C3—C4	1.545 (10)
U1—O6	2.411 (4)	C5—C6	1.517 (10)
U1—O6^i^	2.411 (4)	C5—H5A	0.9900
S1—O3	1.426 (5)	C5—H5B	0.9900
S1—O4	1.437 (6)	C6—C6^i^	1.53 (2)
S1—O2	1.480 (5)	C6—H6A	0.9900
S1—C1	1.844 (7)	C6—H6B	0.9900
F1—C1	1.358 (7)	C7—C8	1.512 (9)
F2—C1	1.347 (8)	C7—H7A	0.9900
F3—C2	1.337 (8)	C7—H7B	0.9900
F4—C2	1.337 (8)	C8—C9	1.505 (10)
F5—C3	1.355 (8)	C8—H8A	0.9900
F6—C3	1.347 (8)	C8—H8B	0.9900
F7—C4	1.329 (12)	C9—C10	1.522 (10)
F8—C4	1.329 (10)	C9—H9A	0.9900
F9—C4	1.289 (12)	C9—H9B	0.9900
O5—C5^i^	1.466 (8)	C10—H10A	0.9900
O5—C5	1.466 (8)	C10—H10B	0.9900
			
O1—U1—O1^i^	179.6 (3)	F6—C3—C4	107.7 (6)
O1—U1—O2	90.9 (2)	F5—C3—C4	107.0 (6)
O1^i^—U1—O2	89.2 (2)	F6—C3—C2	109.5 (5)
O1—U1—O2^i^	89.2 (2)	F5—C3—C2	110.2 (6)
O1^i^—U1—O2^i^	90.9 (2)	C4—C3—C2	115.0 (6)
O2—U1—O2^i^	145.2 (2)	F9—C4—F8	108.9 (8)
O1—U1—O5	90.22 (13)	F9—C4—F7	111.2 (8)
O1^i^—U1—O5	90.22 (13)	F8—C4—F7	107.1 (8)
O2—U1—O5	72.58 (12)	F9—C4—C3	111.1 (7)
O2^i^—U1—O5	72.58 (12)	F8—C4—C3	109.2 (7)
O1—U1—O6	92.67 (18)	F7—C4—C3	109.2 (7)
O1^i^—U1—O6	86.97 (18)	O5—C5—C6	103.9 (6)
O2—U1—O6	71.83 (15)	O5—C5—H5A	111.0
O2^i^—U1—O6	142.95 (15)	C6—C5—H5A	111.0
O5—U1—O6	144.33 (10)	O5—C5—H5B	111.0
O1—U1—O6^i^	86.97 (18)	C6—C5—H5B	111.0
O1^i^—U1—O6^i^	92.67 (18)	H5A—C5—H5B	109.0
O2—U1—O6^i^	142.95 (15)	C5—C6—C6^i^	102.7 (6)
O2^i^—U1—O6^i^	71.83 (15)	C5—C6—H6A	111.2
O5—U1—O6^i^	144.34 (10)	C6^i^—C6—H6A	111.2
O6—U1—O6^i^	71.3 (2)	C5—C6—H6B	111.2
O3—S1—O4	117.7 (4)	C6^i^—C6—H6B	111.2
O3—S1—O2	113.0 (3)	H6A—C6—H6B	109.1
O4—S1—O2	113.6 (3)	O6—C7—C8	104.3 (5)
O3—S1—C1	105.9 (3)	O6—C7—H7A	110.9
O4—S1—C1	104.4 (3)	C8—C7—H7A	110.9
O2—S1—C1	99.8 (3)	O6—C7—H7B	110.9
S1—O2—U1	137.7 (3)	C8—C7—H7B	110.9
C5^i^—O5—C5	110.8 (7)	H7A—C7—H7B	108.9
C5^i^—O5—U1	124.6 (4)	C9—C8—C7	102.9 (5)
C5—O5—U1	124.6 (4)	C9—C8—H8A	111.2
C10—O6—C7	109.4 (5)	C7—C8—H8A	111.2
C10—O6—U1	124.9 (4)	C9—C8—H8B	111.2
C7—O6—U1	124.3 (4)	C7—C8—H8B	111.2
F2—C1—F1	107.7 (6)	H8A—C8—H8B	109.1
F2—C1—C2	110.3 (6)	C8—C9—C10	103.2 (5)
F1—C1—C2	108.0 (5)	C8—C9—H9A	111.1
F2—C1—S1	108.7 (4)	C10—C9—H9A	111.1
F1—C1—S1	108.0 (5)	C8—C9—H9B	111.1
C2—C1—S1	114.0 (5)	C10—C9—H9B	111.1
F3—C2—F4	109.8 (6)	H9A—C9—H9B	109.1
F3—C2—C1	108.9 (6)	O6—C10—C9	105.5 (6)
F4—C2—C1	109.4 (5)	O6—C10—H10A	110.6
F3—C2—C3	108.2 (5)	C9—C10—H10A	110.6
F4—C2—C3	107.7 (6)	O6—C10—H10B	110.6
C1—C2—C3	112.9 (6)	C9—C10—H10B	110.6
F6—C3—F5	107.2 (6)	H10A—C10—H10B	108.8
			
O3—S1—O2—U1	−94.6 (5)	O4—S1—C1—C2	−44.4 (5)
O4—S1—O2—U1	42.7 (5)	O2—S1—C1—C2	−162.0 (5)
C1—S1—O2—U1	153.3 (4)	F2—C1—C2—F3	−161.5 (6)
O1—U1—O2—S1	7.9 (4)	F1—C1—C2—F3	−44.1 (8)
O1^i^—U1—O2—S1	−172.5 (4)	S1—C1—C2—F3	76.0 (6)
O2^i^—U1—O2—S1	−82.1 (4)	F2—C1—C2—F4	78.6 (7)
O5—U1—O2—S1	−82.1 (4)	F1—C1—C2—F4	−164.0 (6)
O6—U1—O2—S1	100.4 (4)	S1—C1—C2—F4	−44.0 (7)
O6^i^—U1—O2—S1	94.0 (5)	F2—C1—C2—C3	−41.3 (8)
O1—U1—O5—C5^i^	−167.8 (4)	F1—C1—C2—C3	76.1 (7)
O1^i^—U1—O5—C5^i^	12.2 (4)	S1—C1—C2—C3	−163.8 (5)
O2—U1—O5—C5^i^	−76.9 (4)	F3—C2—C3—F6	−163.8 (6)
O2^i^—U1—O5—C5^i^	103.1 (4)	F4—C2—C3—F6	−45.2 (7)
O6—U1—O5—C5^i^	−72.9 (4)	C1—C2—C3—F6	75.6 (7)
O6^i^—U1—O5—C5^i^	107.1 (4)	F3—C2—C3—F5	78.6 (7)
O1—U1—O5—C5	12.2 (4)	F4—C2—C3—F5	−162.8 (5)
O1^i^—U1—O5—C5	−167.8 (4)	C1—C2—C3—F5	−42.0 (8)
O2—U1—O5—C5	103.1 (4)	F3—C2—C3—C4	−42.4 (9)
O2^i^—U1—O5—C5	−76.9 (4)	F4—C2—C3—C4	76.2 (8)
O6—U1—O5—C5	107.1 (4)	C1—C2—C3—C4	−163.0 (7)
O6^i^—U1—O5—C5	−72.9 (4)	F6—C3—C4—F9	71.4 (9)
O1—U1—O6—C10	−17.9 (5)	F5—C3—C4—F9	−173.7 (7)
O1^i^—U1—O6—C10	161.8 (5)	C2—C3—C4—F9	−51.0 (10)
O2—U1—O6—C10	−108.0 (5)	F6—C3—C4—F8	−48.6 (10)
O2^i^—U1—O6—C10	74.4 (5)	F5—C3—C4—F8	66.2 (9)
O5—U1—O6—C10	−112.0 (4)	C2—C3—C4—F8	−171.0 (7)
O6^i^—U1—O6—C10	68.0 (4)	F6—C3—C4—F7	−165.5 (7)
O1—U1—O6—C7	147.0 (5)	F5—C3—C4—F7	−50.6 (9)
O1^i^—U1—O6—C7	−33.3 (5)	C2—C3—C4—F7	72.1 (9)
O2—U1—O6—C7	56.9 (4)	C5^i^—O5—C5—C6	−12.6 (4)
O2^i^—U1—O6—C7	−120.8 (5)	U1—O5—C5—C6	167.4 (4)
O5—U1—O6—C7	52.9 (5)	O5—C5—C6—C6^i^	32.4 (9)
O6^i^—U1—O6—C7	−127.1 (5)	C10—O6—C7—C8	−18.3 (7)
O3—S1—C1—F2	−42.9 (5)	U1—O6—C7—C8	174.8 (4)
O4—S1—C1—F2	−167.8 (4)	O6—C7—C8—C9	34.8 (7)
O2—S1—C1—F2	74.6 (5)	C7—C8—C9—C10	−37.8 (8)
O3—S1—C1—F1	−159.5 (5)	C7—O6—C10—C9	−5.4 (7)
O4—S1—C1—F1	75.6 (5)	U1—O6—C10—C9	161.4 (4)
O2—S1—C1—F1	−41.9 (5)	C8—C9—C10—O6	27.0 (7)
O3—S1—C1—C2	80.5 (6)		

Symmetry codes: (i) −*x*, *y*, −*z*+3/2.

### Hydrogen-bond geometry (Å, °)

**Table:**

*D*—H···*A*	*D*—H	H···*A*	*D*···*A*	*D*—H···*A*
C6—H6B···O4^ii^	0.99	2.63	3.57 (1)	159
C7—H7A···O3^iii^	0.99	2.47	3.409 (9)	158

Symmetry codes: (ii) −*x*, −*y*+1, −*z*+1; (iii) *x*, −*y*, *z*+1/2.

## References

[bb1] Alcock, N. W., Kemp, T. J. & Leciejewicz, J. (1993). *Inorg. Chim. Acta*, **203**, 81–86.

[bb2] Altomare, A., Cascarano, G., Giacovazzo, C., Guagliardi, A., Burla, M. C., Polidori, G. & Camalli, M. (1994). *J. Appl. Cryst.* **27**, 435.

[bb3] Berthet, J. C., Lance, M., Nierlich, M. & Ephritikhine, M. (2000). *Eur. J. Inorg. Chem.* pp. 1969–1973.

[bb4] Charpin, P., Lance, M., Nierlich, M., Vigner, D. & Baudin, C. (1987). *Acta Cryst.* C**43**, 1832–1833.

[bb5] Farrugia, L. J. (1997). *J. Appl. Cryst.* **30**, 565.

[bb6] Higashi, T. (1999). *NUMABS* Rigaku Corporation, Tokyo, Japan.

[bb7] MSC/Rigaku (2006). *CrystalStructure* MSC, The Woodlands, Texas, USA, and Rigaku Corporation, Tokyo, Japan.

[bb8] Oldham, S. M., Scott, B. L. & Oldham, W. J. Jr (2006). *Appl. Organomet. Chem.* **20**, 39–43.

[bb9] Rebizant, J., Van den Bossche, G., Spirlet, M. R. & Goffart, J. (1987). *Acta Cryst.* C**43**, 1298–1300.

[bb10] Rigaku (2006). *PROCESS-AUTO* Rigaku Corporation, Tokyo, Japan.

[bb11] Sheldrick, G. M. (1997). *SHELXL97* University of Göttingen, Germany.

[bb12] Thuéry, P., Nierlich, M., Keller, N., Lance, M. & Vigner, J.-D. (1995). *Acta Cryst.* C**51**, 1300–1302.

[bb13] Wilkerson, M. P., Burns, C. J., Paine, R. T. & Scott, B. L. (1999). *Inorg. Chem.* **38**, 4156–4158.

